# Association of apolipoprotein A5 genetic polymorphisms with steroid-induced osteonecrosis of femoral head in a Chinese Han population

**DOI:** 10.1186/s13000-014-0229-1

**Published:** 2014-12-17

**Authors:** Yong Cui, Aihemaiti Kaisaierjiang, Peng Cao, Zhong-Yan Wu, Qing Lv

**Affiliations:** Department of orthopetics, Fifth Affiliated Hospital of Xinjiang Medical University, No.118, Henan West Street, Urumqi, 830011 China

**Keywords:** Apolipoprotein A5, Steroid-induced osteonecrosis, Gene polymorphism

## Abstract

**Background:**

Previous studies suggested that apolipoprotein A5 (ApoA5) genetic polymorphisms (SNPs) may result in lipid metabolism disorders. Therefore, genetic polymorphisms in ApoA5 may be associated with the occurrence of osteonecrosis of femoral head (ONFH).

**Methods:**

We designed a case–control study including 223 patients of osteonecrosis and 201 age- and sex-matched control subjects to analyze the association between ApoA5 polymorphisms and susceptibility of steroid-induced ONFH. We utilized polymerase chain reaction-restriction fragment length polymorphism (PCR-RFLP) method to genotype two SNPs (rs662799 and rs3135506) in *ApoA5* gene.

**Results:**

We found both rs662799 and rs3135506 were associated with the risk of ONFH in codominant, dominant, and recessive model, respectively. Haplotype analyses suggested that T-C haplotype was associated with decreased risk of ONFH, whereas the haplotype C-C was significantly associated with an increased risk of ONFH.

**Conclusion:**

Our study suggested that ApoA5 genetic polymorphisms were associated with susceptibility to ONFH in Chinese population. However, our results need further investigation with large sample size and various populations.

**Virtual Slides:**

The virtual slide(s) for this article can be found here: http://www.diagnosticpathology.diagnomx.eu/vs/13000_2014_229

## Background

Osteonecrosis of femoral head (ONFH) is a kind of orthopedic refractory disease that cellular death happens within femoral head owing to damage of blood supply to the anterior-superior-lateral part of the femoral head [1]. In China, there are approximately 7 million people with ONFH currently. And, new cases reached to 100–200 thousand each year [2]. ONFH is a complex disorder may result from various risk factors such as trauma, alcoholism, coagulation defects, and abnormal lipid metabolism. Recent study suggested that abnormal lipid metabolism is main pathogenesis of osteonecrosis [3]. Hyperlipidemia affects the microcirculation of the femoral head to result in femoral necrosis from multiple links, such as affecting blood coagulation solvent systems, influencing bone fat embolism, and affecting the formation of bone micro-thrombosis [4,5]. ApoA5 is an important apolipoprotein which involved in plasma lipid metabolism. The most significant impact on plasma triglycerides (TG) levels seems to be associated with *ApoA5* gene (ID 116519, OMIM accession number 606368) variants [6,7]. *ApoA5* is located on TG-rich and high density lipoprotein (HDL) particles, enhances the activity of lipoprotein lipase [7,8], and recombinant apoA5 binds to the LDL receptor family members [9]. Previous studies suggested that minor alleles of two SNPs (rs662799 and rs3135506) in *ApoA5* gene were associated with elevated plasma TG levels, regardless of ethnicity and sex [10–13].

In 2007, Hirata et al. [14] found *ApoB* gene polymorphism was associated with osteonecrosis. Subsequently, Wang et al. [15] found -75G > A polymorphism in *ApoAI* gene was associated with osteonecrosis in Chinese population. Recently, Yin et al. also found SNPs in *ApoAI* gene were associated with ONFH in a Chinese Han population [16]. However, the relation between the polymorphisms of *ApoA5* gene and ONFH has not been studied.

In the present study, we designed a case–control study to reveal the relation between *ApoA5* genetic polymorphisms and ONFH in a Chinese population.

### Subjects and methods

#### Ethics

The present study has been performed with the approval of the ethics committee of the 5th Affiliated Hospital of Xinjiang Medical University and was in compliance with the Helsinki Declaration. The informed consents of the study were collected from all the candidate subjects.

#### Subjects

All the patients were selected from January 2001 to December 2013 in the 5th Affiliated Hospital of Xinjiang Medical University. All the patients were consistent with the diagnostic criteria of osteonecrosis proposed in 1995 by Mont et al. [17]. Steroid-induced ONFH was defined by a history of a mean daily dose of ≥ 16.6 mg or highest daily dose of 80 mg of predinosolone equivalent within 1 year prior to the development of symptoms or radiological diagnosis in asymptomatic cases [18–20]. All the patients were confirmed by clinical diagnosis, double hip X-ray image, CT scan or MRI examination. We included 223 patients with steroid-induced ONFH (case group, 121 men, 102 women; mean age: 42.27 ± 15.71 years) and 201 patients who did not develop steroid-induced ONFH (control group, 112 men, 89 women; mean age: 43.33 ± 15.02 years) following steroid administration for the present study. The clinical characteristics of patients in case and control groups were summarized in Table 1.

**Table 1:** **Characteristics of the participants**

**Groups**	**N**	**Age (years)**	**Gender (M/F)**	**BMI Kg/m** ^**2**^	**GLU (mmol/L)**	**TG (mmol/L)**	**TC (mmol/L)**	**HDL-C (mmol/L)**	**LDL-C (mmol/L)**
ONFH group	223	42.27 ± 15.71	121/102	23.7 ± 3.5	5.8 ± 1.4	2.5 ± 1.1	5.3 ± 2.4	1.5 ± 0.7	2.9 ± 1.6
Control group	201	43.33 ± 15.02	112/89	23.9 ± 3.2	5.7 ± 1.2	1.4 ± 1.3	4.6 ± 2.1	1.4 ± 0.8	2.2 ± 1.4
*P*		0.703	0.654	0.261	0.352	<0.001	0.001	0.184	0.007

Note: BMI = Body mass index; GLU = Glucose; TC = Total cholesterol; LDL-C = Low-density lipoprotein-cholesterol; LDL-C = High-density lipoprotein-cholesterol; TG = Triglycerides.

#### SNPs selection and genotyping

Two SNPs (rs662799 and rs3135506), which were reported to be associated with plasma lipid level, in the *ApoA5* gene were selected in this study. Blood samples were collected using a standard venipuncture technique and EDTA-containing tubes. DNA was extracted from peripheral vein blood leukocytes using a whole blood genome extraction kit (Beijing Boiteke Corporation, Beijing, China). SNPs rs662799 and rs3135506 were genotyped using PCR-RFLP as described in details elsewhere [21–23]. Briefly, polymerase chain reaction (PCR) was performed in a volume of 25 ml containing 200 ng of genomic DNA. The amounts of Mg^2+^, dNTP, and DNA polymerase (Bangalore Genei, India) used in each reaction were 1.5 mM, 200 mM, and 1 U, respectively. The thermal cycles started with 94°C for 4 min and were followed by 35 cycles of 94°C for 30 s, 55°C for 30 s, and 72°C for 30 s. A total volume of 20 ul containing 20 U endonuclease was added directly to the PCR product and digested at 37°C overnight. After electrophoresis, the digested products were visualized on a 3% polyacrylamide gel with ethidium bromide staining.

### Statistical analysis

Data were analyzed using SPSS 17.0 software package (Chicago, IL, USA).The genotype and allele frequencies were calculated by direct counting method. The differences of genotype and allele distributions between case and control groups were compared using χ2 test, OR value and its 95% CI was calculated according to χ2 test. Normality was assessed by plotting the residuals. Statistical significance was set at p <0.05.

## Results

### Characteristics of participants

The characteristics of 223 patients and 201 control subjects were shown in Table 1. After the statistical analysis, there were no significant differences in the distribution of age, sex, body mass index (BMI), GLU, and HDL-C between the two groups. However, there were significant differences in TG, TC and LDL-C between these two groups.

### Hardy-Weinberg equilibrium

The genotype distributions were in Hardy-Weinberg equilibrium in both case group and control group (both P > 0.05, data not shown).

### Genotype and allele frequency distributions

These two SNPs had a strong association with ONFH in all genetic models (As shown in Table 2).To determine the extent of linkage disequilibrium (LD) between the two polymorphisms, standardized LD coefficient D’ and r^2^ were calculated (D’ = 0.65, and r^2^ = 0.12).

**Table 2:** **Genotypic distribution of ApoA5 polymorphisms**

**SNP**	**Model**	**Controls**	**Patients**	**OR (95% CI)**	**P value**
rs662799	Codominant				
	TT	192 (95.51)	146 (65.2)	1	
	TC	3 (1.49)	30 (13.2)	10.33 (2.54-52.77)	<0.001
	CC	6 (3.0)	47 (21.6)	15.32 (4.12-55.67)	<0.001
	Dominant				
	TT	192 (95.51)	146 (65.2)	1	
	TC + CC	9 (4.49)	77 (34.8)	12.11 (4.33-36.21)	<0.001
	Recessive				
	TT + TC	195 (97.0)	176 (78.4)	1	
	CC	6 (3.0)	47 (21.6)	13.33 (3.21-44.05)	<0.001
rs3135506	Codominant				
	CC	176 (87.51)	159 (70.98)	1	
	CG	22 (11.0)	32 (14.29)	1.43 (0.82-3.76)	0.088
	GG	3 (1.49)	33 (14.73)	12.11 (3.66-67.12)	<0.001
	Dominant				
	CC	176 (87.51)	159 (70.98)	1	
	CG + GG	25 (12.49)	65 (29.02)	3.01 (1.98-6.33)	<0.001
	Recessive				
	CC + CG	196 (98.51)	191 (85.27)	1	
	CC	3 (1.49)	33 (14.73)	12.22 (2.12-50.76)	<0.001

### Haplotype analysis of rs662799 and rs3135506

To examine the combined effect of the two SNPs (rs662799 and rs3135506) in the *ApoA5* gene, we constructed 4 haplotypes between the two SNPs. As shown in Table 3, T-C haplotype was associated with decreased risk of ONFH (P = 0.003, OR = 0.605 95% CI: 0.430 ~ 0.852), whereas the haplotype C-G (P = 0.002, OR = 2.223; 95% CI: 1.144 ~ 3.361) was significantly associated with an increased risk of ONFH. No significant association was observed between the haplotype T-G and C-C and ONFH.

**Table 3:** **Distribution of haplotypes**

**Haplotype**	**ONFH**	**Control**	***P***	**OR (95% CI)**
T-C	0.109	0.247	0.003	0.605 [0.430 ~ 0.852]
T-G	0.213	0.195	0.199	1.455 [0.796 ~ 4.905]
C-C	0.143	0.152	0.117	0.936 [0.811 ~ 1.982]
C-G	0.122	0.060	0.002	2.223 [1.144 ~ 3.361]

### ApoA5 genetic polymorphisms and lipids levels

In addition, age and sex adjusted intergenotypic variations in lipid levels with respect to *apoA5* polymorphisms have been summarized in Table 4. Rare C allele of rs662799 and G allele of rs3135506 carriers were associated with higher levels of TG in ONFH patients (both *P* < 0.05).

**Table 4:** **Comparison of lipids levels between each genotype**

		**rs662799**		**rs3135506**			
**Parameters**	**Groups**	**TT**	**TC/CC**	**CC**	**CG/GG**	P*	P**
TC (mg/dl)	Case	5.0 ± 2.7 (n = 146)	5.2 ± 2.5 (n = 77)	5.2 ± 2.6 (n = 159)	5.0 ± 2.2 (n = 65)	0.125	0.442
	Control	4.4 ± 2.1 (n = 192)	4.9 ± 2.4 (n = 9)	4.6 ± 2.3 (n = 176)	4.8 ± 2.1 (n = 25)	0.112	0.314
LDL-C (mg/dl)	Case	2.6 ± 1.2	2.9 ± 1.3	2.7 ± 1.1	2.6 ± 1.3	0.109	0.197
	Control	2.3 ± 1.3	2.5 ± 1.2	2.4 ± 1.1	2.5 ± 1.3	0.221	0.453
HDL-C (mg/dl)	Case	1.8 ± 0.9	1.6 ± 0.7	1.6 ± 0.8	1.7 ± 0.8	0.198	0.3381
	Control	1.7 ± 1.0	1.6 ± 0.8	1.6 ± 1.0	1.4 ± 0.8	0.332	0.143
TG (mg/dl)	Case	2.1 ± 1.4	2.7 ± 0.8	2.0 ± 1.0	2.8 ± 0.9	0.013	0.024
	Control	1.7 ± 0.8	1.6 ± 1.1	1.8 ± 0.9	1.5 ± 1.1	0.435	0.174

Note: TC = Total cholesterol; LDL-C = Low-density lipoprotein-cholesterol; LDL-C = High-density lipoprotein-cholesterol; TG = Triglycerides; *P value for rs662799; **P value for rs3135506.

## Discussion

In the present study, we found that in patients with ONFH, C allele of rs662799 and G allele of rs3135506 in apoA5 gene were significantly higher than that in the control group, the T-C haplotype frequency was significantly lower than that in the control group (*P* <0.0001) and C-G haplotype was common in the control group. This is the first study to clarify the relation between *ApoA5* polymorphism and ONFH.

The abnormal lipid metabolism and intravascular coagulation composed the main pathogenesis of osteonecrosis [3]. Hyperlipidemia affected the microcirculation of the femoral head resulting in femoral necrosis from multiple links, such as affecting blood coagulation solvent systems, influencing bone fat embolism, and affecting the formation of bone micro-thrombosis [21,22]. Many evidences showed that *ApoA5* gene is associated with serum lipid levels [10]. Recent findings indicate that *ApoA5* could also influence cholesterol homeostasis and probably could play a role in hypertriglyceridemia [11]. In our study, we found polymorphisms of *ApoA5* gene are associated with TG levels. The carriers with mutant alleles have higher levels of TG in the ONFH patients. Therefore, we consider the mechanism of the association of *ApoA5* polymorphisms with ONFH may resulting from lipids level changes caused by *ApoA5* genetic polymorphism.

Although we found a positive association between *ApoA5* polymorphisms and ONFH, the present study was limited by the relatively small sample size. This may have led to weak statistical significance and wide CIs when estimating odds ratios. In addition, we did not perform functional study of these two SNPs, which may be another limitation of our study.

## Conclusion

In conclusion, this study showed that *ApoA5* polymorphism may be associated with ONFH in Han Chinese population.
